# Label-free photoacoustic microscopy for *in-vivo* tendon imaging using a fiber-based pulse laser

**DOI:** 10.1038/s41598-018-23113-y

**Published:** 2018-03-19

**Authors:** Hwi Don Lee, Jun Geun Shin, Hoon Hyun, Bong-Ahn Yu, Tae Joong Eom

**Affiliations:** 1 Advanced Photonics Research Institute, Gwangju Institute of Science and Technology, 123 Cheomdan-gwagiro, Buk-gu, Gwangju 61005 South Korea; 2 School of Electrical Engineering and Computer Science, Gwangju Institute of Science and Technology, 123 Cheomdan-gwagiro, Buk-gu, Gwangju 61005 South Korea; 3 Department of Biomedical Sciences, Chonnam National University Medical School, 160 Baekseo-ro, Dong-gu, Gwangju 61469 South Korea; 4 Present Address: Advanced Photonics Research Institute, Gwangju Science and Technology, Gwangju, 61005 South Korea

## Abstract

Tendons are tough, flexible, and ubiquitous tissues that connect muscle to bone. Tendon injuries are a common musculoskeletal injury, which affect 7% of all patients and are involved in up to 50% of sports-related injuries in the United States. Various imaging modalities are used to evaluate tendons, and both magnetic resonance imaging and sonography are used clinically to evaluate tendons with non-invasive and non-ionizing radiation. However, these modalities cannot provide 3-dimensional (3D) structural images and are limited by angle dependency. In addition, anisotropy is an artifact that is unique to the musculoskeletal system. Thus, great care should be taken during tendon imaging. The present study evaluated a functional photoacoustic microscopy system for *in-vivo* tendon imaging without labeling. Tendons have a higher density of type 1 collagen in a cross-linked triple-helical formation (65–80% dry-weight collagen and 1–2% elastin in a proteoglycan-water matrix) than other tissues, which provides clear endogenous absorption contrast in the near-infrared spectrum. Therefore, photoacoustic imaging with a high sensitivity to absorption contrast is a powerful tool for label-free imaging of tendons. A pulsed near-infrared fiber-based laser with a centered wavelength of 780 nm was used for the imaging, and this system successfully provided a 3D image of mouse tendons with a wide field of view (5 × 5 mm^2^).

## Introduction

Tendons are tough, flexible, and ubiquitous tissues that connect muscle to bone and transfer the force that is generated by muscle movement to the bone^1^. For example, muscle contraction pulls the tendon and transfers force to the connected bone. Thus, tendons play an important role in muscle-bone control of the body. Tendon injuries are a common musculoskeletal injury that affect 7% of all patients and are involved in up to 50% of sports-related injuries in the United States^2–4^.

Various imaging modalities can be used to obtain tendon images, and both magnetic resonance imaging (MRI)^5–8^ and sonography^8,9^ are used clinically for tendon imaging with non-invasive and non-ionizing radiation. In addition, optical coherence tomography (OCT) has been used for tendon imaging in preclinical studies^10,11^.

In the MRI sequences, normal tendons have low signal intensity because the alignment of water and collagen molecules in the tendon structure generates dipole interactions that considerably shorten the T_2_ relaxation time to 1–2 ms^12–14^. In addition, tendons generate a characteristic angle-dependent MRI signal. The T_2_ begins to increase when the angle between the tendon and the magnetic field is above 20° and maximized around 55° (the “magic angle” phenomenon)^14–18^. Collagen orientation of the tendon at this “magic angle” shows higher MRI signal intensity, depending on the type of sequence and the echo time. Therefore, the orientation change of tendon fibers can cause signal degradation and lead to a misinterpreted diagnosis. This problem can be reduced using a long echo time and stronger magnetic field, which provides a better signal-to-noise ratio (SNR) and resolution^19^.

During sonographic tendon imaging, sound waves reflect back to the ultrasound transducer from the collagen fiber bundles of the tendon. This generates echogenic parallel lines in the longitudinal plane and multiple echogenic dots in the transverse plane^20^. When imaging tendons, the ultrasound beam should be perpendicular to the collagen fibers, and even a 2° deviation can eliminate the sonographic image and potentially lead to a misinterpreted diagnosis^20^. This artifact is called anisotropy and is unique to the musculoskeletal system. Therefore, great care should be taken during sonographic imaging of tendons that are not parallel to the skin’s surface.

An OCT system is a noninvasive optical imaging technique for performing high-resolution (micrometer) cross-sectional *in-vivo* imaging. The intensity OCT image can provide structural tomographic information of the biological tissues, although it is difficult to classify the boundary of tissues when they have a low difference of refractive index. Tendons are surrounded with other tissues, such as the dermis and muscle, which are closely located and have a small difference of refractive index. Therefore, the boundaries of the tendons are not clearly shown in an OCT image of the musculoskeletal system^10^.

The polarization sensitive OCT (PS-OCT) system, which is one of the functional OCT modalities, is conceived to image the polarization properties of biological tissue based on polarization-sensitive detection. High birefringence of the collagen bundle and fibrillose structure of the tendon makes a difference in the propagation constant of each polarized light that passes through the biological tissue. The PS-OCT system can measure the tiny difference of the propagation properties by measuring the phase difference of the polarization components and obtain functional OCT images of the tendon tissue^10,11^. However, the PS-OCT system is challenging to apply to *in-vivo* tendon tomography because of its imaging depth limitation. A strong turbid and randomly polarized media (e.g., the epidermis and dermis) interrupt to maintain the polarization information obtained from the deep tendon structure.

The photoacoustic (PA) imaging system is a recently developed and promising hybrid microscopic imaging technique^21–26^. During PA imaging, a short-pulse laser irradiates the target tissues, which have optical absorption in the wavelength of the laser, undergo both spatial and temporal thermo-elastic expansion, and generates broadband ultrasound waves. The PA images can be obtained with signal filtering and data processing of the detected ultrasound signals. Many types of PA imaging systems have been developed for vascular imaging, blood oxygen saturation (SO_2_) mapping, and brain activity mapping by using endogenous absorption contrast of biological chromophores, such as hemoglobin, melanoma, and lipid^21–27^. In addition, many researches have been showed the potential for clinical application by using point-of-care system, handheld probe, volumetric temperature mapping and all-optical ultrasound transducer array^28–31^.

In the present study, we investigated the optical absorption property of tendon and developed *in-vivo* label-free PA microscopy (PAM) system using high absorption wavelength of the tendon. Tendons have a high density of type 1 collagen arranged in a cross-linked triple-helical formation compared to other tissues, with a dry mass of 65–86% collagen and 1–2% elastin, which is embedded in a proteoglycan-water matrix^32^. The collagen provides a striking endogenous absorption contrast in the near-infrared (NIR) spectrum^33–35^. We used a 780-nm pulsed laser matched with the absorption property of the tendon. The fiber laser is used for the light source of the PAM, which can enhance stability, accessibility, selectivity, and acquisition time of the imaging system^36^. We successfully obtained *in-vivo* label-free tendon PA images of mice with a wide field of view (FOV; 5 × 5 mm^2^).

## Results

### Characteristics of the tendons

**Figure 1:**
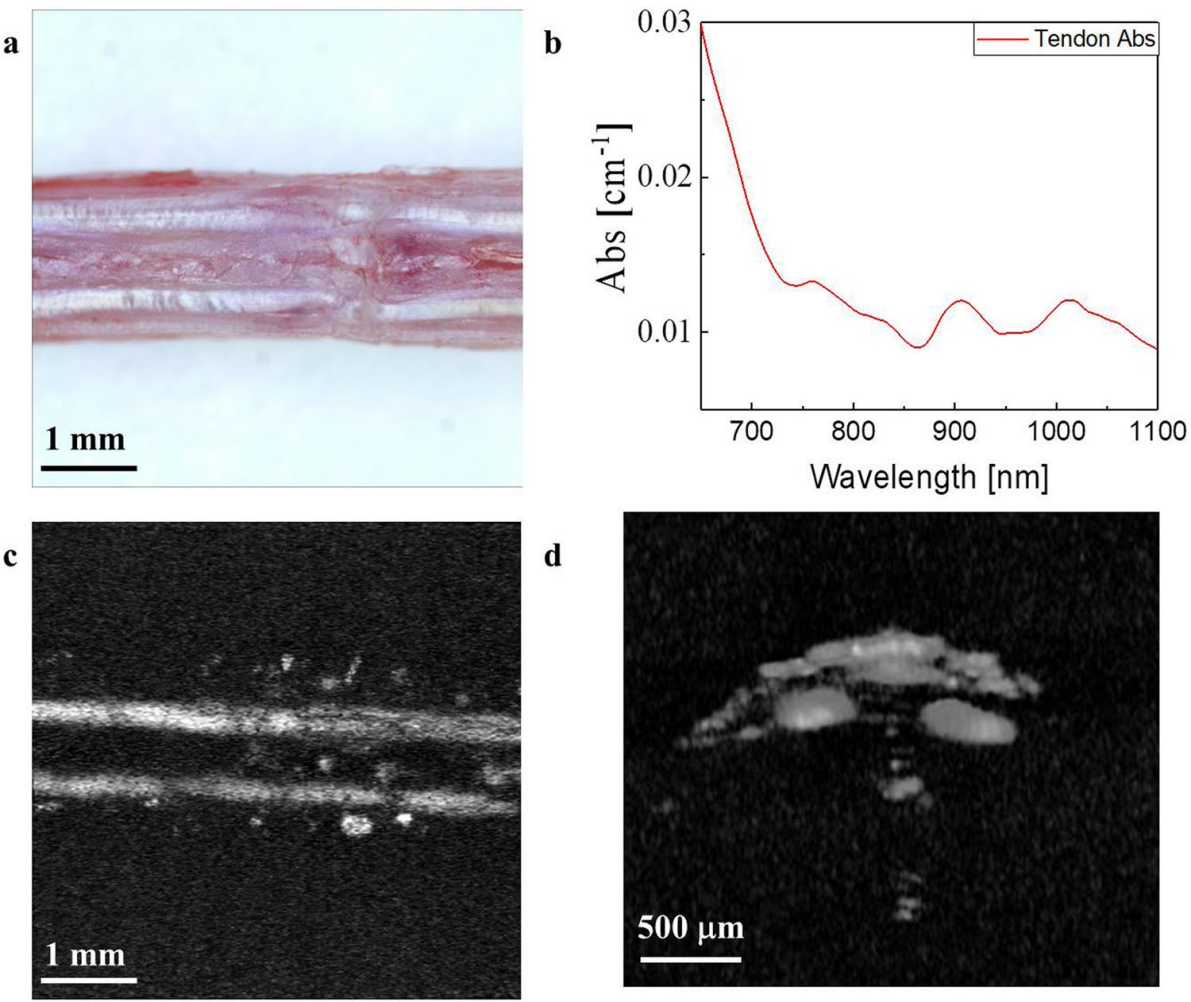
(**a**) Photograph of a mouse tail with the skin removed. (**b**) Absorption (Abs) spectrum of the mouse tendon. (**c**) Enface and (**d**) depth-resolved *in vivo* photoacoustic microscopy images of a mouse tail.

Figure 1a shows a mouse’s tail after skin removal, and Fig. 1b shows the absorption spectrum of tendon in the NIR spectrum. The NIR spectral region (approximately 650–950 nm) or “optical window” has deep photon penetration due to relatively high transparency and reduced scattering^37,38^. Some critical chromophores exhibit different absorption properties in the NIR spectrum, such as oxy-hemoglobin, deoxy-hemoglobin, cytochrome oxidase, lipid, melanin, and collagen^34^. The absorption coefficient of the collagen is almost 30 times higher than other chromophore such as Hb, HbO_2_ and lipid at 780 nm region^34^ (Supplementary Figure S1). To verify that tendon mainly consists of the collagen, we obtained the absorption spectrum of the tendon after extracting from mouse tail (See method). As shown in Fig. 1b, the tendon has an absorption spectrum similar to that of collagen because the tendon predominantly consists of collagen^33,34^. In addition, the mature tendons are poorly vascularized which means low blood vessels in the tendon region. A nutrition of tendon is more reliant on synovial fluid diffusion than vascular perfusion^39^. Although the absorption coefficient of collagen is higher at the shorter wavelength than 780 nm, the absorption of the melanin becomes higher and close to value of collagen at this region. Similarly, the absorption coefficient of lipid becomes higher at the longer wavelength region^34^. Therefore, the wavelength of 780 nm, which has higher tendon absorption than other chromophores, was selected for the label-free tendon PAM imaging system.

The PAM system for *in-vivo* tendon imaging was performed using the tail of a nude mouse (Balb/C, 4–5 weeks old, approximately 20 g). Figure 1c,d show PA images of a mouse tail for enface and depth-resolved image, respectively, and that the PA signals were selectively generated from the tendon region. The OCT images were obtained with the same sample for both *ex vivo* (after skin removal) and *in-vivo* (Supplementary Figure S2). As we expected, the tendon regions are not clearly shown in the OCT image because this modality lacks selectivity for imaging tendons.

**Figure 2:**
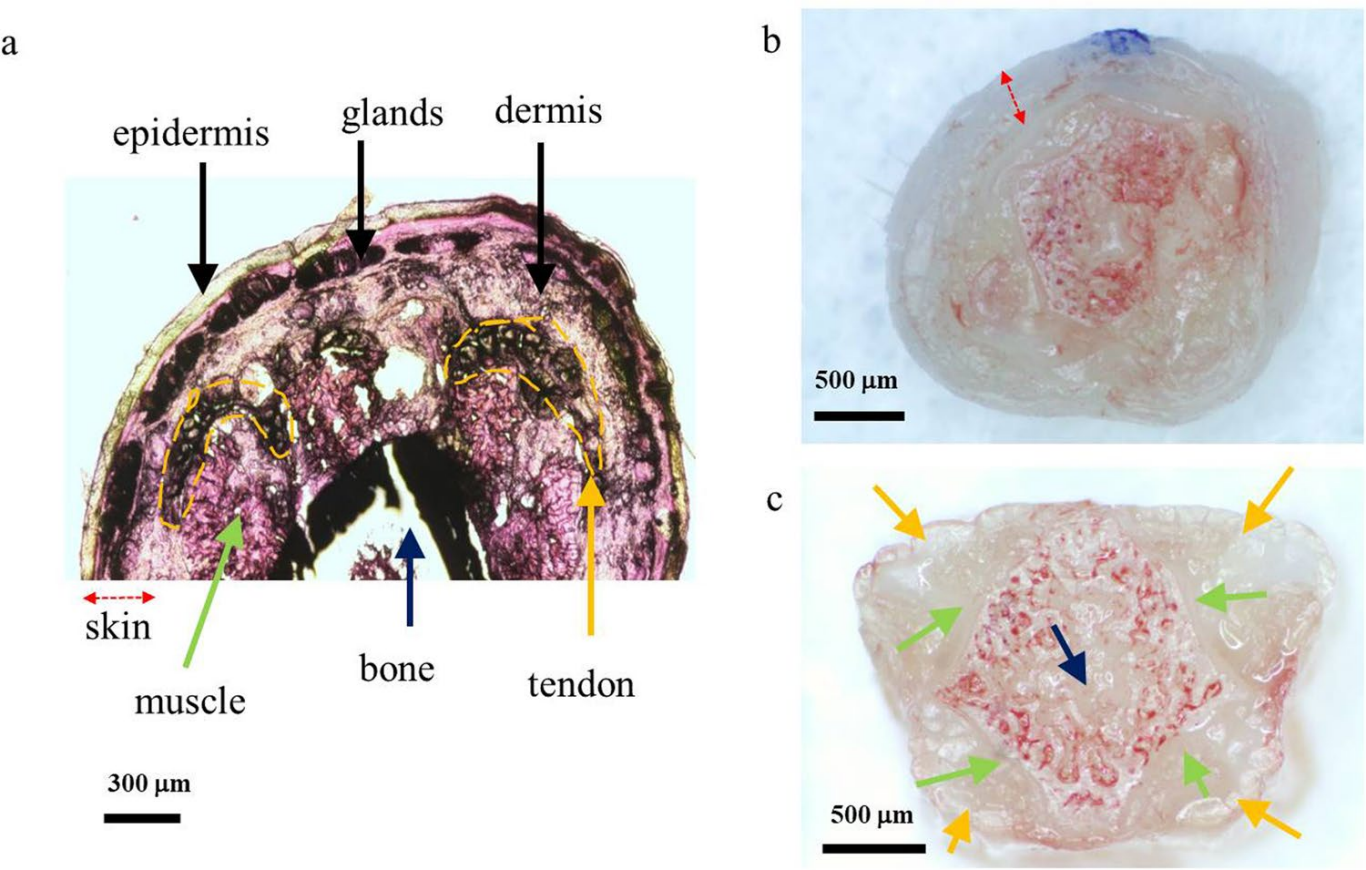
(**a**) A stained cross-section image of a mouse tail. (**b**) Cross-sectional photograph of a mouse tail. (**c**) Cross-sectional photograph of a mouse tail after skin removal.

Figure 2a shows a stained image of a mouse tail at the dorsal region. Figure 2b,c show cross-sectional images of a mouse tail before and after skin removal, respectively. As shown in Fig. 2(a–c), four tendon bundles are located between the dermis layer of the skin and muscles in the mouse tail (two in the dorsal region and two in the ventral region). Compared to Figs 1a and 2(a–c), the proposed PAM system appears to provide high-contrast tendon images without labeling. Unlike other commercial tendon imaging modalities (e.g., MRI or sonography), the PAM system provides depth-resolved 3D images of the tendon with minimizing angle dependency (Supplementary Video S1). The measured FOV was 5 × 5 mm^2^ along the X and Y axes, and the axial and lateral system resolutions were 42.83 μm and 78.58 μm, respectively. The total acquisition time of the system is 500 seconds which is relatively slow than other systems because we used slow voice-coil stage for high-load sample arm.

### The 1560-nm pulsed fiber laser

**Figure 3:**
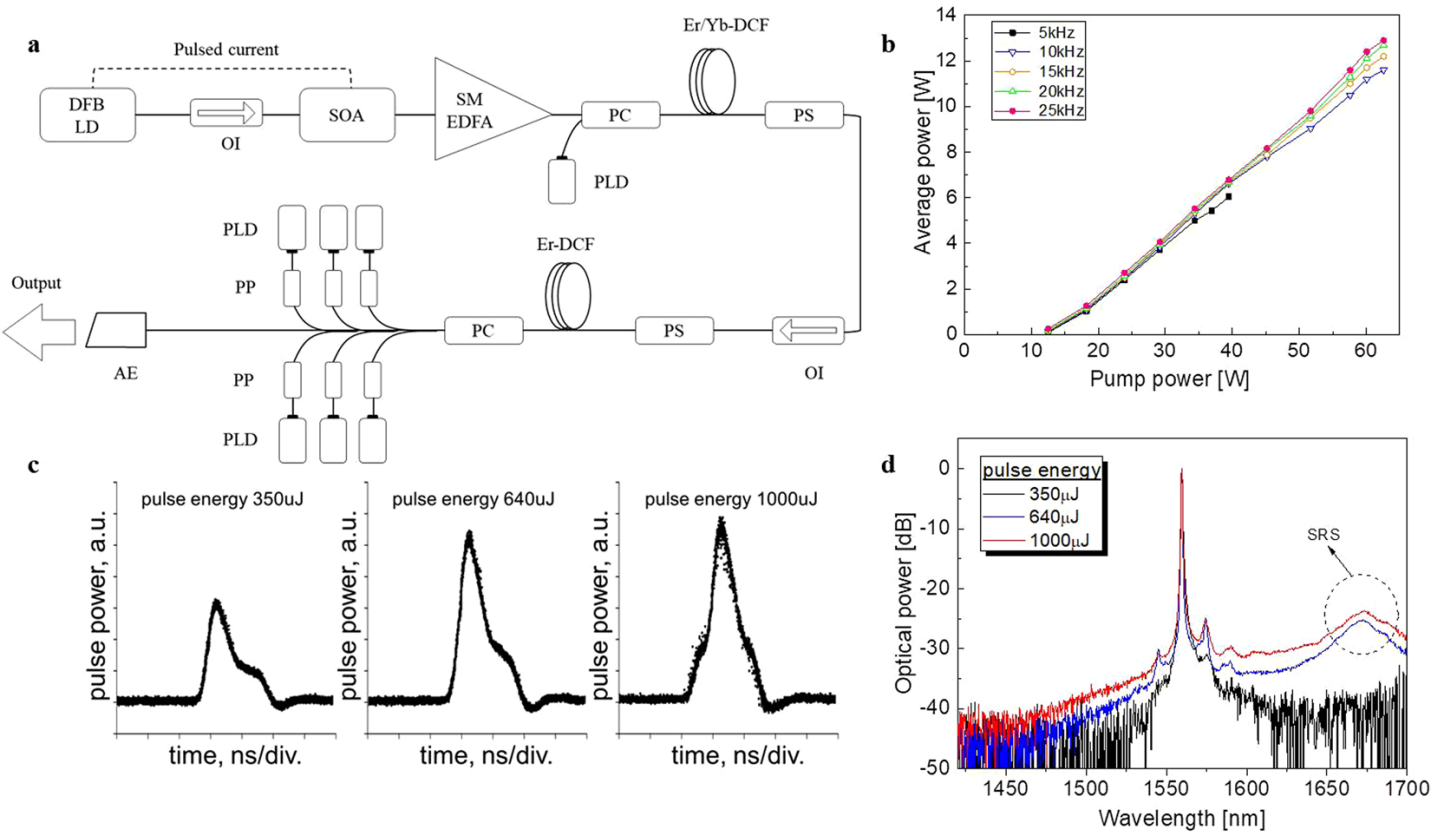
(**a**) Schematic of the proposed pulsed all-fiber source. (**b**) Achieved output average power of the pulse source at pulse repetition rates of 5, 10, 15, 20, and 25 kHz as a function of pump power coupled to Er-DCF. (**c**) Waveform traces and optical spectra of output pulses with three different pulse energies at a pulse repetition rate of 10 kHz. (**d**) Optical spectrum of output pulses with a maximum peak power of 450 kW at a pulse repetition rate of 25 kHz. OI: optical isolator; PLD: pump laser diode; PC: pump/signal combiner; PS: pump stripper; PP: pump protector; AE: angled endcap; Er-DCF: Er-doped double-clad fiber; Er/Yb-DCF: Er/Yb co-doped double-clad fiber; SOA: semiconductor optical amplifier; SM EDFA: semiconductor optical amplifier; DFB: distributed feedback; LD: laser diode.

For the proposed PAM system, we used a 1560 nm pulsed fiber laser, which lacks a high-gain medium at 780 nm. Figure 3a shows the schematic for the laser source, which is an all-optical fiber system that consists of a direct modulated laser diode (LD)/semiconductor optical amplifier (SOA) combination seeder, dual-stage single mode Er-doped fiber pre-amplifier (SM EDFA), an Er/Yb co-doped double-clad fiber (Er/Yb-DCF) booster amplifier, and Er-doped double-clad fiber (Er-DCF) power amplifier.

A 1560 nm distributed feedback LD followed by an SOA was used as the seeder. The seed pulses are delivered to the dual-stage SM EDFA, which is core-pumped by two 980 nm SM pump laser diodes (PLDs) through wavelength division multiplexer couplers. The pre-amplified pulses have a pulse energy that ranges from 2.2 μJ at 25 kHz to 4.2 μJ at 5 kHz and a spectrum width of <0.05 nm when approximately 1.8 ns seed pulses are injected at a pulse repetition rate (PRR) of 5–25 kHz. The pre-amplified pulses are further amplified in a 1.2 m multimode Er/Yb-DCF with a 23 μm core diameter. The amplified pulse energy is restricted to approximately 10 μJ at all PRRs, based on the peak power handling limit of the optical isolator after the booster amplifier. The pulses have a narrower pulse width (1.1–1.3 ns) than the seed pulse width, which is attributable to the pulse-shaping effects of gain saturation in the fiber amplifiers^40^. The power amplifier is based on a large-core 6.5 m multimode Er-DCF with a 70 μm core diameter and an approximately 1.6 dB/m peak cladding absorption at 980 nm.

Figure 3b shows the total output power of the final pulse source at PRRs of 5–25 kHz, against a pump power that is coupled to the Er-DCF. A maximum power of up to 12.9 W was achieved at a PRR of 25 kHz under a pump power of 62.6 W. The output power was stable, with root-mean-square fluctuation of <1% when the average power was monitored for 30 minutes. The measured power intrinsically includes background amplified spontaneous emission (ASE), which can be easily calculated by subtracting the corresponding pulse average power (pulse energy × PRR) from the measured output power. For example, the measured pulse energy was 462 μJ when the source emitted the maximum output power of 12.9 W at a PRR of 25 kHz, which corresponds to a background ASE of 1.35 W or 10.5% of the output power.

Figure 3c,d show the recorded pulse waveforms and optical spectra with three different pulse energies at a PRR of 10 kHz. Substantial waveform distortion and stimulated Raman scattering (SRS) growth were observed at a pulse peak power of >450 kW, which corresponds to an approximate pulse energy of >450 μJ. The source eventually generated pulses with a maximum peak power of 450 kW at a PRR of 25 kHz under the maximum pump power of 62.6 W. The pulses had a pulse width of approximately 0.8 ns, 3 dB linewidth of about 0.4 nm, and a little SRS (Supplementary Figure S3(a)). Similar pulses with the same peak power could be obtained at other PRRs (5–20 kHz) with the reduced pump power.

### Photoacoustic microscopy system with the 780 nm pulsed laser

**Figure 4:**
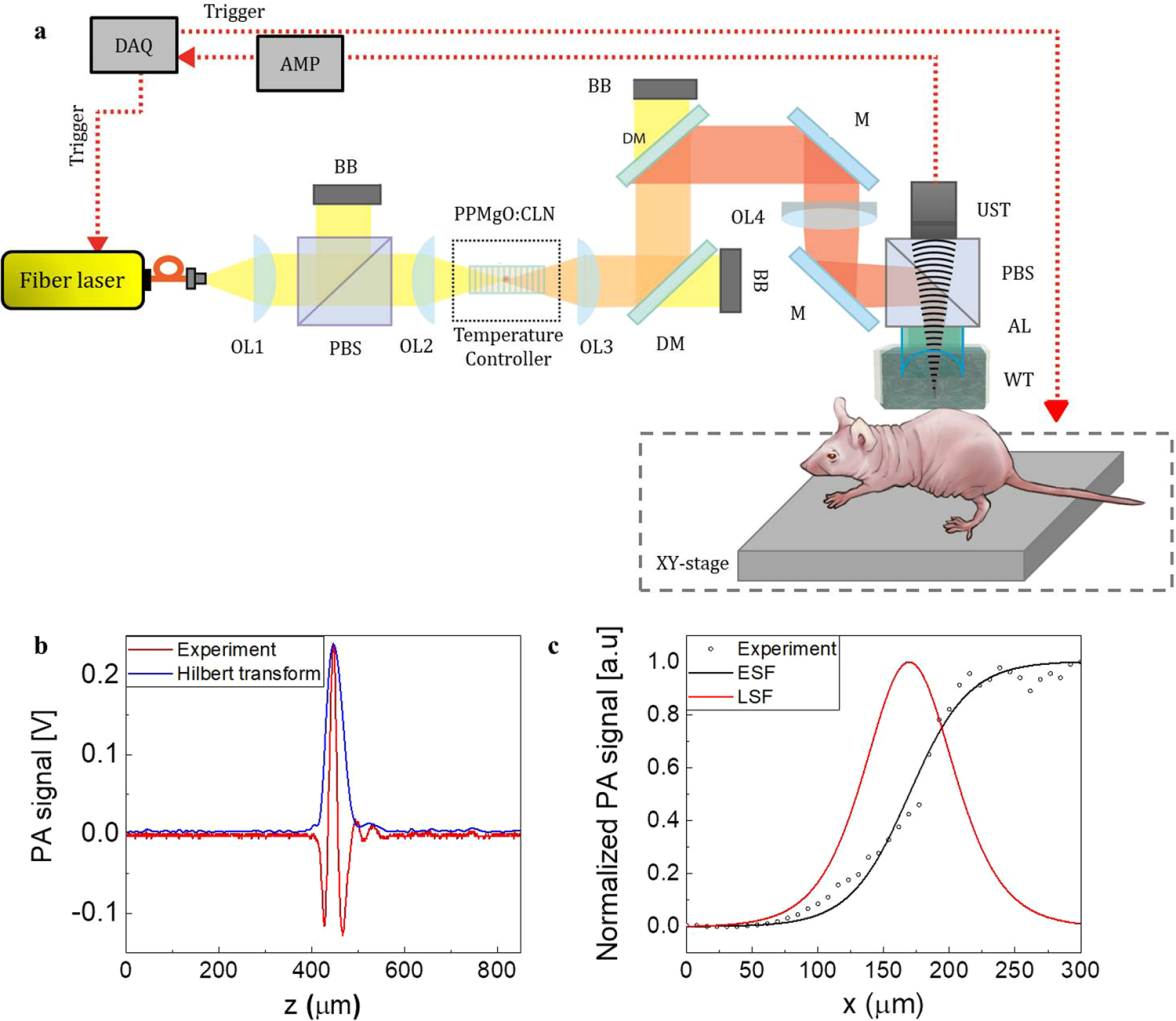
(**a**) Experimental setup of the *in vivo* PAM system. (**b**) Axial and (**c**) lateral resolutions of the PAM system. OL: Optical lens; PBS: polarization beam splitter; BB: beam block; DM: dichroic mirror; M: mirror; Samp: sample; WT: water tank; AL: acoustic lens; UST: ultrasound transducer; PAM: photoacoustic microscopy.

Figure 4a shows the experimental schematic for the label-free PAM system for *in-vivo* tendon imaging. To exploit the high optical absorption of tendons, a high-power infrared fiber laser and second harmonic generation (SHG) method were used for the optical source^41^. The pulsed fiber laser had a center wavelength of 1560 nm, an output power of 4.5 W, a pulse width of approximately 0.8 ns, PRR of 10 kHz, and maximum energy of 450 μJ before the SHG module.

For the SHG module, a polarization beam splitter was used to match the polarization states between the input beam and a crystal. An anti-reflection coated plano-convex lens with a focal length of 75 mm was used to focus the 1560 nm laser onto the crystal (20 mm PPMgO: CLN), and an additional plano-convex lens with a 75 mm focal length was used for light collimation. Two dichroic mirrors were used to separate the beams with wavelengths of 780 nm and 1560 nm. The measured maximum output power of the SHG beam was 500 mW, and the relative pulse energy was 50 μJ after the two dichroic mirrors (Supplementary Figure S3(b)). The output NIR pulse laser was focused using a 780 nm coated doublet lens with a focal length of 75 mm, and the confocal position of an ultrasound transducer (UST) was determined using an acoustic lens with a focal length of 11 mm (Supplementary Figure S4). The PA signals generated from the target sample were obtained by UST, which has a central frequency of 30 MHz and a bandwidth of 20.04 MHz.

We developed acoustic-resolution photoacoustic microscopy (AR-PAM) system for tendon imaging because the 1560 nm pulsed laser shows multi-modal properties. The AR-PAM comprises a focused UST and a weakly focused light beam, therefore, a lateral resolution of the system is lower than optical-resolution PAM (OR-PAM). However, the AR-PAM can detect more deeply located target than OR-PAM which is more suitable for tendon imaging.

To verify the performance of the proposed PAM system with the 780 nm laser source, we prepared a tungsten wire with a thickness of 30 μm and a sharp blade for axial and lateral resolution, respectively. As shown in Fig. 4b, the axial resolution was estimated to be 42.83 μm. A theoretical axial resolution was determined based on the bandwidth of the ultrasonic transducer, as follows if the impulse response of the UST has a Gaussian envelope: Ra ≈ 0.88 × c/B = 39.45 μm, where Ra is the axial resolution, c is the speed of sound, B is the acoustic −6 dB bandwidth, and the center frequency of the UST is 30 MHz. The SNR of the PAM system was 37 dB. The lateral resolution was obtained using the line spread function, which is the first-order derivative of the edge spread function, as shown in Fig. 4c. The full-width half-maximum of the line spread function indicated that the lateral resolution was 78.58 μm. As the 1560 nm fiber laser is based on the multi-mode fiber amplification for high pulse energy over 450 μJ, the beam profile of the 780 nm laser shows multi-modal properties, which is difficult to achieve with high lateral resolution. For fast axis scanning, the voice coil motor was applied to the system, and a slow axis scanning was achieved by using the linear servo stage. In our system, these high load scanning stages and high current driver for the PAM system could cause unwanted ambient noise with periodic strength over time. We removed the noise using a quasi-periodic noise removal method (Supplementary Figure S5)^42^. The PAM data were obtained with 2000 × 500 × 500 voxels per volume dataset matched with the scanning range over 7.5 mm × 5 mm × 5 mm.

### Label-free *in-vivo* tendon imaging

**Figure 5:**
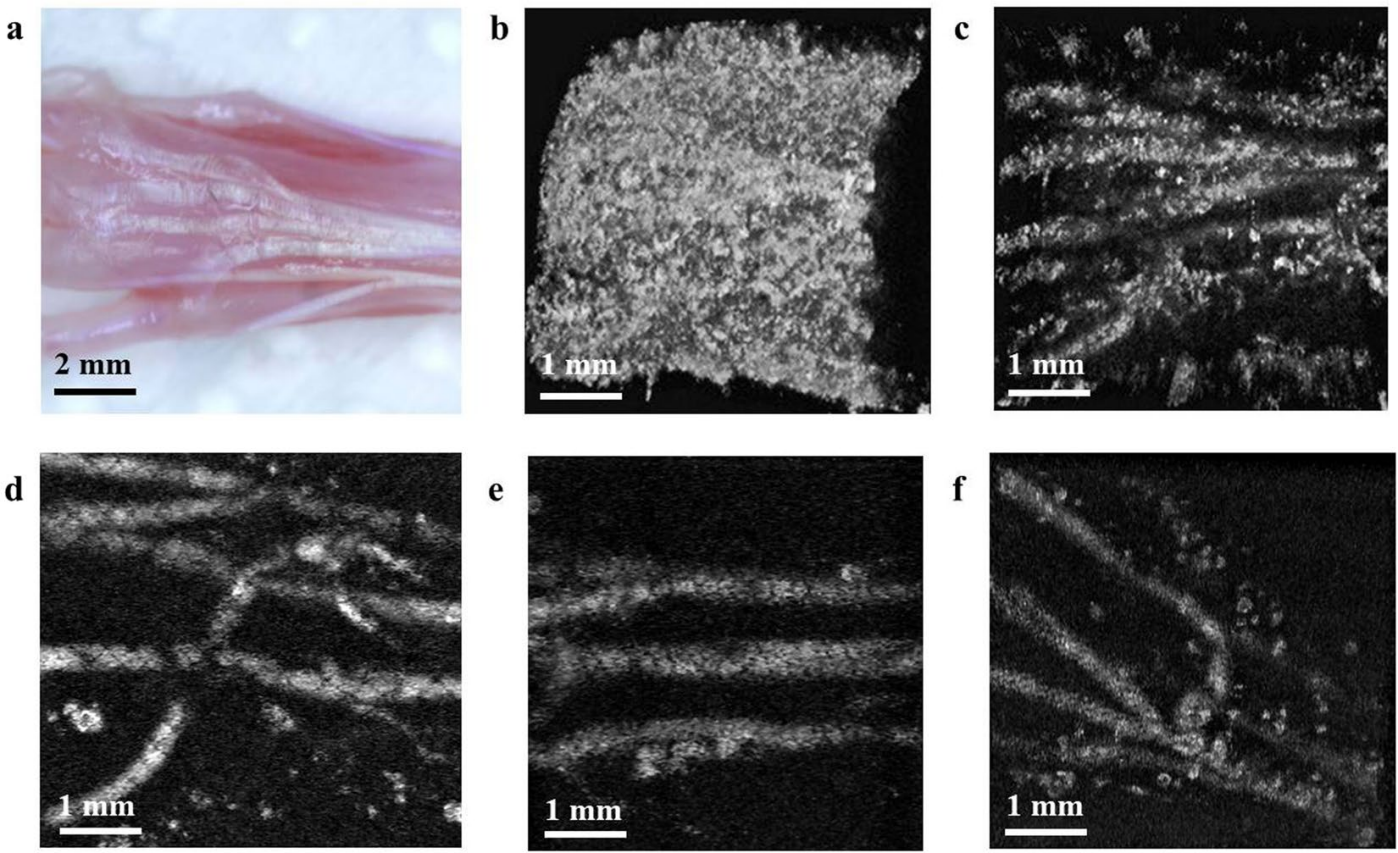
(**a**) Photograph of a mouse paw with skin removed. Top view of 3-dimensional rendered PAM images focused on the (**b**) skin and (**c**) skin layer and tendon layer; (**b**) and (**c**) were obtained simultaneously. Label-free PAM image of the (**d**) paw, (**e**) ankle, and (**f**) paw/ankle focused on the tendon layer. PAM: photoacoustic microscopy.

In the present study, we generated *in-vivo* images of the mouse paw to provide a model with potential clinical application. Figure 5a shows a photograph of the paw region after removal of the skin, and Fig. 5(b–f) show the various label-free tendon PA images with the mouse paw in various orientations. When the illuminated laser beams are focused on the skin surface region, we observed unexpected PA signals from the skin area, as shown in Fig. 4b. Collagens in the dermis layer can contribute to generating the PA signals when the laser beams are tightly focused at the skin (glands, Fig. 2a). To obtain a high-sensitive tendon PA image, as shown in Fig. 4c, additional image processes were required to segment PA images between dermis and tendon layers. The tendons were located under the dermis of the mouse skin (separated by several hundred microns), which suggests that the PA signals from different tissues can be classified according to the depth. We controlled the focal depth of the beam around the tendon depth region to maximize the signal amplitude from the tendon region and to simplify the image segmentation process. As shown in Fig. 5(d–f), the PA images were obtained with high sensitivity to tendons. Since the proposed tendon imaging system does not have any angle dependency between the illuminated laser beam and the sample’s structural orientation, the obtained PA images did not show any signal fluctuation from the angle and position dependency of the tendon orientation change unlike sonography and MRI^14–17,20^. Therefore, we successfully reconstructed the 3D *in-vivo* tendon images of the mouse paw (Supplementary Video S2), ankle, and paw/ankle in a wild mice model study.

## Discussion

We described the label-free PAM system for *in-vivo* tendon imaging using a 780 nm fiber-based pulsed laser. The proposed method was demonstrated using a tail, paw, and ankle in a mouse model. The PA image of the *in vivo* study demonstrated that the structural details in the region of a tendon could be clearly classified from other structures (e.g., the epidermis, dermis, glands, and muscle). This modality is based on the light absorption property of the collagen and has been shown to select the tendon structural information without additionally labeling material injection and the angle dependency between the illuminating laser beams and the tendon orientations.

High-resolution imaging of tendons plays an important role in orthopedics because many patients have a tendon injury, especially up to 50% of sports-related persons. We expect that our PAM system, which is highly sensitive for tendon imaging, can provide an objective indicator for tendon injury. Although the PA signals are generated from the dermis layer, the tendon region can be easily classified by the image depth level or by controlling the focal position. Further study of the following is needed to improve the system: (1) use of a high-numerical-aperture lens in the sample probe and a single-mode fiber laser to improve lateral resolution, (2) enhancement of the imaging speed using a UST array or high-speed beam scanning methods, (3) enhancement of the imaging depth by using photoacoustic tomographic regime, and (4) monitoring of the healing process to determine any changes in the injured tendons.

## Methods

### Absorption spectrum of the tendons

Diffuse reflectance spectra of prepared tendons were recorded at 650–1100 nm using a UV-VIS spectrophotometer (UV-3600, Shimadzu) with an integrating sphere. To obtain the absorption spectrum, we extracted the tail tendon after sacrificing a single nude mouse. The tendon is polished to powder state before measurement. The calibration process for the diffuse reflectance measurements was performed using BaSO_4_ as the reference material. The reflectance spectra were subsequently converted into absorbance using the Kubelka-Munk function^43^.

### The 1560-nm fiber laser

The fiber laser uses a master oscillator power amplifier configuration that is composed of a direct modulated LD/SOA combination seeder and Er-doped fiber amplifier chains. The LD and SOA are synchronized and current-modulated with a metal-oxide-semiconductor field-effect transistor differential switching driver. By adjusting the time delay between the LD and SOA, the seeder can generate pulses with a shorter duration than the pulse duration of the driving current. The Er/Yb DCF is forward-pumped using a 980 nm multimode PLD through a multimode pump/signal combiner. The Er-DCF is backward-pumped by six 980 nm multi-mode LDs through a (6 + 1) × 1 signal/pump combiner, which provides a maximum coupled pump power of 62.6 W. A pump stripper is used to eliminate residual pump power, and the pump protectors are inserted to prevent failure of the PLDs because of the backward high-peak power pulses. The pulse peak power can be determined from the pulse energy and pulse waveform by equating the pulse energy to the integral under the pulse waveform (roughly corresponding to the pulse width)^44^.

The pulse waveform was measured using a high-speed InGaAs detector (Thorlabs Inc.), and the peak waveform values were proportional to the calculated pulse peak power until the peak power reached approximately 450 kW in the full range of the PRR. For a peak power over 450 kW, the recorded waveform was distorted from the true pulse shape because the high-peak power in the fiber induced significant SRS at an approximate wavelength of 1670 nm (the detector has poor responsivity at wavelengths of >1650 nm)^45^.

### Second harmonic generation

The nonlinear crystal (PPMgO: CLN, HC Photonics) for the SHG had a length of 20 mm, width of 1.4 mm, and height of 1 mm, with an operating temperature of 112.8 °C for generating a wavelength of 780 nm. The input beam was perpendicularly polarized using a polarization beam splitter (PBS) entered into the crystal, and the related polarized output beam was generated at a wavelength of 780 nm. The pulse energy of the 1560-nm laser after PBS (before it was entered into the crystal) is about 200 μJ, and the obtained SHG efficiency is approximately 25% for our system.

### Photoacoustic microscopy system

The polarization beam splitter (47–779, Edmund Optics), prism (32–544, Edmund Optics), and acoustic lens (45–913, Edmund Optics) were immersed in the ultrasound transducer (30 MHz, Olympus NDT) for ultrasound coupling. The focusing lens (doublet lens, Thorlabs, Inc.) had a focal length of 75 mm and was located above the prism. The acoustic and optical axes were maintained in confocal alignment along the entire FOV, which provides a high SNR (Supplementary Figure S4(b)). The trigger signals from the fiber laser initiate the scanning stages and PA signal acquisition. Fast and slow axes scanning were performed using a voice coil stage (MGV84-AVM90-30, Akribis Systems, Inc.) and a linear DC-servo stage, respectively. The PA signals were amplified using an RF amplifier (ZFL-500LN-BNC, 24 dB gain, Mini-Circuit) and converted into digital signals using a digitizer at 100 MS/s (ATS9350, Alazartech, Inc.). The data acquisition software was developed in LabVIEW (National Instrument) with 2000 × 500 × 500 voxels per volume dataset. The data analysis was performed using MATLAB (MathWorks, Inc.). As the tendons have enough optical absorption at the 780 nm wavelength, we obtained PA images of the tendons without averaging the multiple acquired signals. The final 3D volumetric images were rendered using commercial software Amira (FEI Visualization Sciences Group). The 3D images have a FOV of 5 × 5 mm^2^ at an acquisition depth of 7.5 mm. The effective depth limit of the sample is approximately 2 mm because of the high absorption at the dermis and tendon.

### Data processing

The quasi-periodic noise removal method is focused on the removal of signals that have a strong spectral amplitude. The filtering result is obtained by 2-dimensional (2D) median filter in the frequency domain and can be expressed as:

1 $${\hat{X}}_{ij}=\{\begin{array}{c}MED({X}_{ij}),\,if\frac{{X}_{ij}}{MED({X}_{ij})}\ge {\rm{\Theta }}\,{\rm{and}}\,{\rm{not}}\,{\rm{j}}={\rm{i}}=0\\ \quad \quad \quad \quad \quad \,{X}_{ij}\,,\,otherwise\end{array}$$

where $X$ is the spectral amplitude of the signal, $\hat{X}$ is the resultant spectrum, $(i,j)$ is the coordinate of the signal, $MED\,(\ldots )$ is the 2D median value calculation, and ${\rm{\Theta }}$ is the threshold value^42^.

The periodic noises with strong spectral amplitude over the threshold in the frequency domain are replaced by the median value of the adjacent spectral signal, and the signals that do not exceed the threshold are preserved.

### Histologic analysis

Tail tissues of the nude mice were placed in 2% paraformaldehyde in phosphate-buffered saline for 30 minutes before mounting in Tissue-Tek OCT compound (Fisher Scientific) and flash frozen in liquid nitrogen. Frozen samples were cryosectioned (20 µm per slice) and stained with hematoxylin and eosin. The stained tissues were captured by a microscope slide scanner (3D HISTECH, Ltd.).

### Optical coherence tomography system

A swept-source OCT system was used to obtain tomographic *in vivo* and *ex vivo* images of a mouse tail. The central wavelength of the swept source (Axsun, Inc.) was 1310 nm, and the incident power at the sample was 2 mW. The OCT system has an axial resolution of 8.9 μm and a frame rate of 80 frames/s for 500 A-lines per frame^46^. All volumetric data (500 × 500 × 700 pixels) were rendered using the commercial software Amira (FEI Visualization Sciences Group).

### Experimental animals for *in vivo* imaging

Male adult Balb/C mice (4–5 weeks old, approximately 20 g) were used for the *in vivo* imaging. All experimental procedures were performed in accordance with the protocols that were approved by the Animal Care and Ethics Committees of the Gwangju Institute of Science and Technology (GIST-IACUC-2015-89). The pulsed laser illumination energy on the skin surface was below the ANSI limitation of 28.9 mJ/cm^2^ for a wavelength of 780 nm^47^.

## Electronic supplementary material

Supplementary Video S1

Supplementary Video S2

Supplementary Information

## References

[1] Thorpe CT (2016). Anatomical heterogeneity of tendon: Fascicular and interfascicular tendon compartments have distinct proteomic composition. Sci. Rep..

[2] Kannus P, Natri A (1997). Etiology and pathophysiology of tendon ruptures in sports. Scand. J. Med. Sci. Sports.

[3] Kannus P (1997). Etiology and pathophysiology of chronic tendon disorders in sports. Scand. J. Med. Sci. Sports.

[4] Sharma P, Maffulli N (2005). Tendon injury and tendinopathy: Healing and repair. J. Bone Joint Surg. Am..

[5] Schweitzer ME, Karasick D (2000). MR imaging of disorders of the achilles tendon. AJR. Am. J. Roentgenol..

[6] Clavero JA (2002). MR imaigng of ligament and tendon injuries the fingers. Radiographics.

[7] Wang X, Rosenberg ZS, Mechlin MB, Schwei Yao J (2005). M. E. Normal Variants and Diseases of the Peroneal Tendons and Superior Peroneal Retinaculum: MR Imaging Features. Radiographics.

[8] Robinson P (2009). Sonography of CommonTendon Injuries. AJR. Am. J. Roentgenol..

[9] Dong Q, Fessell DP (2009). Achilles Tendon Ultrasound Technique. AJR. Am. J. Roentgenol..

[10] Rashidifard C, Vercollone C, Martin S, Liu B, Brezinski ME (2013). The application of optical coherence tomography in musculoskeletal disease. Arthritis.

[11] Lee SW, Jeong HW, Kim B-M (2010). High-speed spectral domain polarization-sensitive optical coherence tomography using a single camera and an optical switch at 1.3 μm. J. Biomed. Opt..

[12] GuinelFilho H, Du J, Pak BC (2009). Quantitative characterization of the achilles tendon in cadaveric specimens: T1 and T2* measurements using ultrashort-TE MRI at 3 T. Am. J. Roentgenol..

[13] Gold GE, Wren T, Nayak K, Nishimura D, Beaupre G (2001). *In vivo* short echo time imaging of achilles tendon. Proc. Intl. Soc. Mag. Reson. Med..

[14] Du J, Chiang AJ, Chung CB (2010). Orientational analysis of the achilles tendon and enthesis using an ultrashort echo time spectroscopic imaging sequence. Magn. Reson. Imaging.

[15] Erickson SJ (1991). Effect of tendon orientation on MR imaging signal intensity: A manifestation of the “magic angle” phenomenon. Radiology.

[16] Oatridge A, Herlihy AH, Thomas RW (2001). Magnetic resonance: Magic angle imaging of the achilles tendon. The Lancet..

[17] Fullerton GD, Rahal A (2007). Collagen structure: The molecular source of the tendon magic angle effect. J. Magn. Reson. Imaging.

[18] Peto S, Gillis P, Henri VP (1990). Structure and dynamics of water in tendon from NMR relaxation measurements. Biophys. J..

[19] Magee T, Williams D (2006). 3.0-T MRI of the supraspinatus tendon. Am. J. Roentgenol..

[20] Crass JR, van de Vegte GL, Harkavy LA (1988). Tendon echogenicity: *ex vivo*study. Radiology.

[21] Yao J (2015). High-speed label-free functional photoacoustic microscopy of mouse brain in action. Nature Methods.

[22] Hai P, Yao J, Maslov KI, Zhou Y, Wang LV (2014). Near-infrared Optical-resolution photoacoustic microscopy. Opt. Lett..

[23] He Y (2016). *In vivo* label-free photoacoustic flow cytography and on-the-sopt laser killing of single circulating melanoma cells. Sci. Rep..

[24] Kim JY, Lee C, Park K, Han S, Kim C (2016). High-speed and high-SNR photoacoustic microscopy based on a galvanometer mirror in non-conducting liquid. Sci. Rep..

[25] Zhang HF, Maslov K, Stoica G, Wang LV (2006). Functional photoacoustic microscopy for high-resolution and noninvasive *in vivo* imaging. Nature Biotechnol..

[26] Yao J (2016). Multiscale photoacoustic tomography using reversibly switchable bacterial phytochrome as a near-infrared photochromic probe. Nature Methods.

[27] Piao Z (2015). High speed intravascular photoacoustic imaging with fast optical parametric oscillator laser at 1.7 mm. Appl. Phys. Lett..

[28] Berg PJVD, Daoudi K, Moens HJB, Steenbergen W (2017). Feasibility of photoacoustic/ultrasound imaging of synovitis in finger joints using a point-of-care system. Photoacoustics.

[29] Park K (2017). Handheld Photoacoustic MicroscopyProbe. Sci. Rep..

[30] Landa FJO, Dean-Ben XL, Sroka R, Razansky D (2017). Volumetric Optoacoustic Temperature mapping in Photothermal Therapy. Sci. Rep..

[31] Alles EJ (2017). A reconfigurable all-optical ultrasound transducer array for 3D endoscopic imaging. Sci. Rep..

[32] Kannus P (2000). Structure of the tendon connective tissue. *Scand*. J. Med. Sci. Sports.

[33] Taroni P, Comelli D, Pifferi A, Torricelli A, Cubeddu R (2007). Absorption of collagen: effect on theestimate of breast composition and related diagnostic implications. J. Biomed. Opt..

[34] Scholkmann F (2014). review on continuous wave functional near-infrared spectroscopy and imaging instrumentation and methodology. Neouroimage.

[35] Wilson RH, Nadeau KP, Jaworski BF, Tromberg BJ, Burkin AJ (2015). Review of short-wave infrared spectroscopy and imaging methods for biological tissue characterization. J. Biomed. Opt..

[36] Aytac-Kipergil E (2016). B. Development of a fiber laser with independently adjustable properties for optical resolution photoacoustic microscopy. Sci. Rep..

[37] Weissleder R (2001). A clearer vision for *in vivo* imaging. Nature Biotechnol..

[38] Frangioni JV (2003). *In vivo* near-infrared fluorescence imaging. Curr. Opin. Chem. Biol..

[39] Fenwick SA, Hazleman BL, Riley GP (2001). The vasculature and its role in the damaged and gealing tendon. Arthritis Res..

[40] Vu KT (2006). Adaptive pulse shape control in a diode-seeded nanosecond fibre MOPA system. Opt. Express.

[41] Franken P, Hill A, Peters C, Weinreich G (1961). Generation of optical harmonics. Phys. Rev. Lett..

[42] Aizenberg IN, Butakoff C (2002). Frequency domain medianlike filter for periodic and quasi-periodic noise removal. Proc. SPIE—Image Processing: Algorithms Systems.

[43] Lindberg JD, Laude LS (1974). Measurement of the Absorption Coefficient of Atmospheric Dust. Appl. Opt..

[44] Desmoulins S, Teodoro FD (2008). High-gain Er-doped fibre amplifier generating eye-safe MW peak-power, mJ-energy pulses. Opt. Express.

[45] Agrawal, G. P. Nonlinear fibre optics. *Academic Press* **8** (2001).

[46] Hwang H (2013). *In Vivo* 3D Meibography of the Human Eyelid Using Real Time Imaging Fourier-Domain OCT. Plos One.

[47] Laser Institute of America, *American National Standards for Safe Use of Lasers in Health Care*, **ANSI Z136**.**3- 2011**, 45–46 (2012)

