# Supplementary material for: Label-free photoacoustic microscopy for in-vivo tendon imaging using a fiber-based pulse laser
Source: Sci Rep. 2018 Mar 19;8:4805. doi: 10.1038/s41598-018-23113-y (PMC5859263; doi:10.1038/s41598-018-23113-y)
Supplement: Supplementary file 3 — Supplementary Information [file 41598_2018_23113_MOESM3_ESM.pdf]

Label-free photoacoustic microscopy for *in-vivo* tendon imaging using fiber-based pulse laser

Hwi Don Lee[1], Jun Geun Shin[2], Hoon Hyun[2], Bong-Ahn Yu[1*], Tae Joong Eom[1*]

[1]*Department of Medical System Engineering, Gwangju Institute of Science and Technology, 123 Cheomdan-gwagiro, Buk-gu, Gwangju 61005, South Korea*
[2]*Electrical Engineering, California Institute of Technology, 1200 E California Boulevard, Pasadena, California, 91125, USA*
[3]*Gwangju Institute of Science and Technology, Advanced Photonics Research Institute, 123 Cheomdan-gwagiro, Buk-gu, Gwangju 61005, South Korea*

[*]Correspondence and requests for materials should be addressed to B-.A.Y. (bayu@gist.ac.kr) or T.J.E. (eomtj@gist.ac.kr).

Supplementary Figures

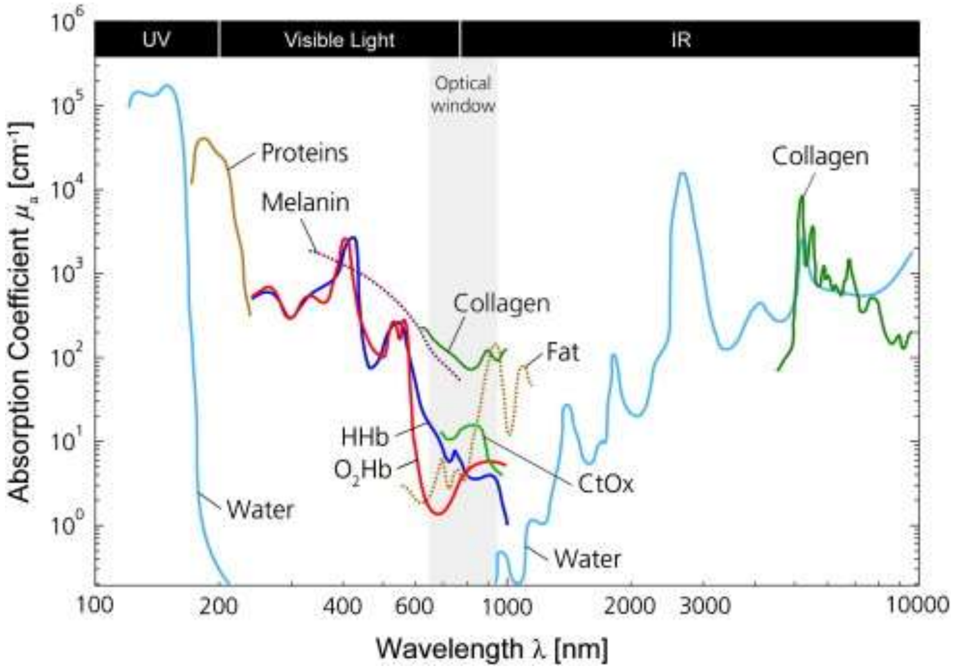

Supplementary Figure S1 | Absorption spectra (natural logarithm base!) for different chromophores present in human tissue. Shown are the spectra for O_2Hb, HHb, proteins, water, collagen, fat and cytochrome oxidase (CtO_x) in the region from 100 nm to 10,000 nm. The spectra are given with respect to the specific concentration in mM. (Reference 34)

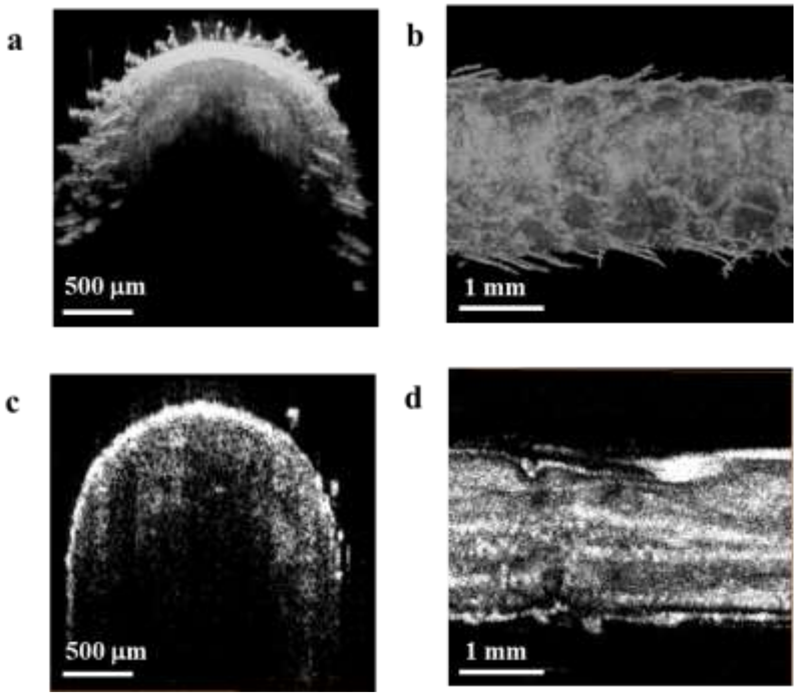

Supplementary Figure S2 | three-dimensional optical coherence tomography image of mouse tail (a) *in-vivo* depth-resolved (b) *en face* (c) *ex-vivo* depth-resolved (d) en-face

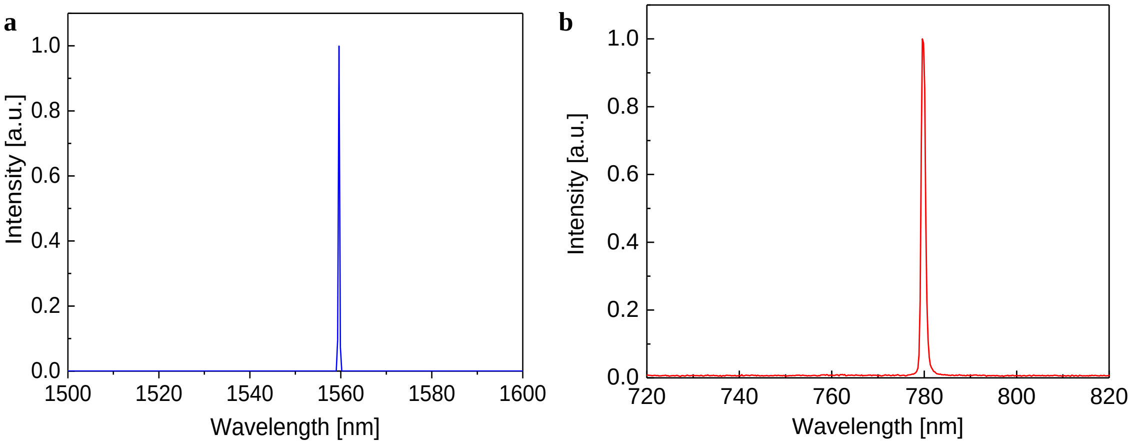

Supplementary Figure S3 | (a) Optical spectrum of output pulse with maximum peak power of 450 kW at PRR of 25 kHz (b) Optical spectrum of output pulse after second harmonic generation

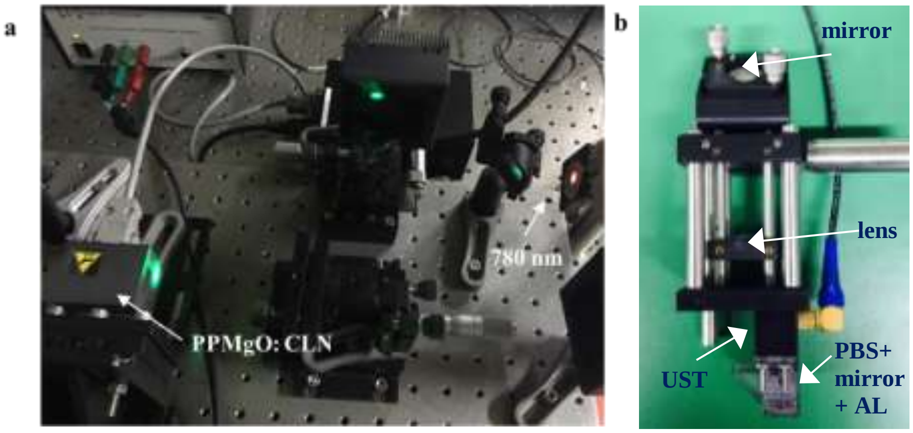

Supplementary Figure S4 | Photography of (a) second harmonic generation module and (b) sample arm. UST: ultrasound transducer ; PBS : polarization beam splitter; AL: acoustic lens ; red arrow : adjusting range of lens

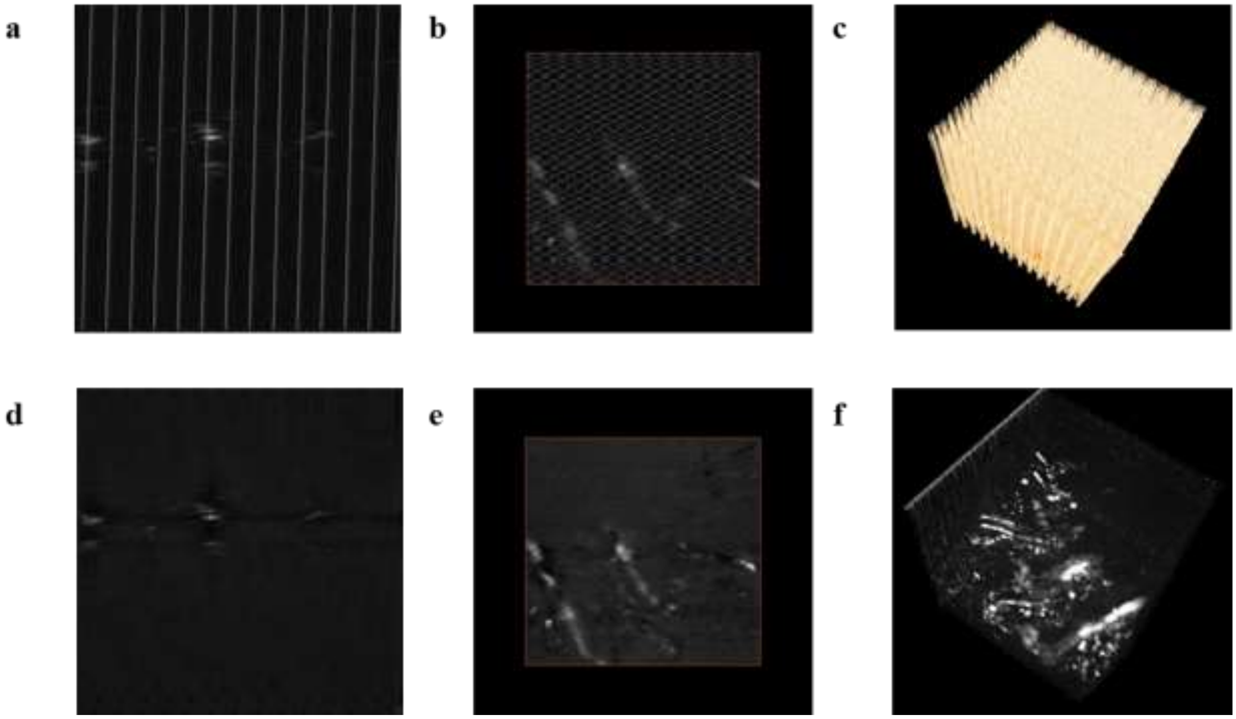

Supplementary Figure S5 | PAM images before quasi-periodic noise removing (a) depth-resolved (b) enface (c) 3D rendering. PAM images after quasi-periodic noise removing (d) depth-resolved (e) enface (f) 3D rendering.

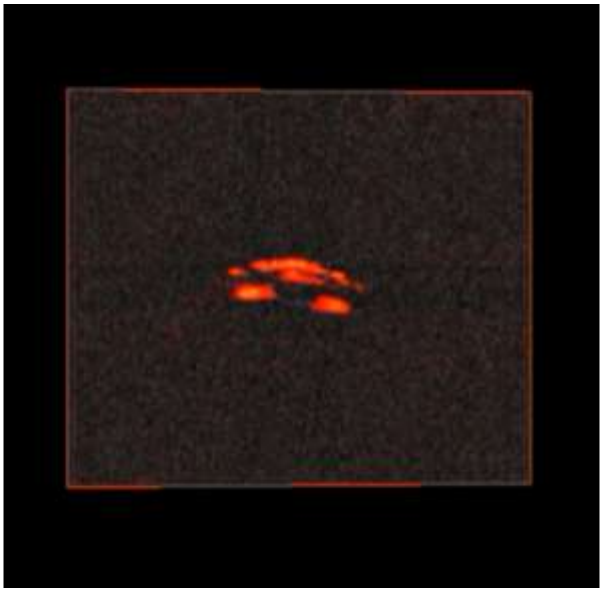

Supplementary Video S1 | *In-vivo* three-dimensional tendon imaging movie of the mouse tail. The movie is processed by commercial software, Arima.

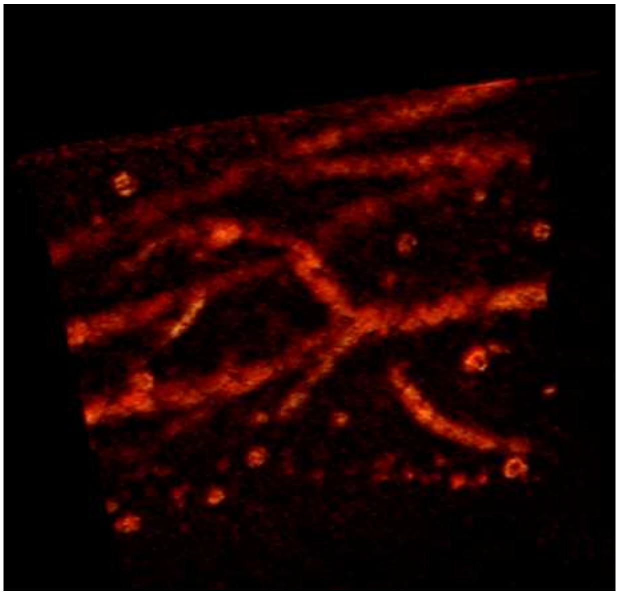

Supplementary Video S2 | *In-vivo* three-dimensional tendon imaging movie of the mouse paw. The movie is processed by commercial software, Arima.
